# Spatiotemporal Evolution of Global Greenhouse Gas Emissions Transferring via Trade: Influencing Factors and Policy Implications

**DOI:** 10.3390/ijerph17145065

**Published:** 2020-07-14

**Authors:** Zhangqi Zhong, Xu Zhang, Weina Gao

**Affiliations:** 1 School of Economics, Zhejiang University of Finance & Economics, Hangzhou 310018, China; zzhongz@zufe.edu.cn (Z.Z.); fantuanaiian@foxmail.com (X.Z.); 2 Center for Regional Economy & Integrated Development, Zhejiang University of Finance & Economics, Hangzhou 310018, China; 3 The New Types Key Think Tank of Zhejiang Province, China Research Institute of Regulation and Public Policy, Zhejiang University of Finance & Economics, Hangzhou 310018, China; 4 China Institute of Regulation Research, Zhejiang University of Finance & Economics, Hangzhou 310018, China

**Keywords:** economic production, environment governance, energy structure, spatial econometric regression models, multi-regional input-output models

## Abstract

Global climate change caused by greenhouse gas emissions (GHGs) from anthropogenic activities have already become the focus of the world. A more systematic and comprehensive analysis on the factors influencing the changes of global GHGs transferring via trade have not been fully discussed. To this end, employing spatial econometric regression models and multi-regional input-output models, this paper reveals factors influencing the GHGs transferring via trade changes in 39 major economies, so as to develop the relevant GHGs reduction policies. The results indicate that regions with the highest net outflow of GHGs transferring via trade are primarily Russia and Canada, and the adverse effects of promoting GHGs reduction on the national economy could be avoided by these regions owing to trade relations. Additionally, factors influencing the changes in GHGs transferring via trade have significant spatial autocorrelation, and population size and energy structure exert significant spatial spillover effects on the changes in the GHGs transferring via trade. On this basis, this paper suggests that one more effective way to prevent trade from the rigorous demands of environmental governance measures while preserving the economic benefits of international trade may be to facilitate cooperation between countries on GHGs mitigation. Further, we articulate more balanced environment governance policies, including conducting the sharing of advanced energy technologies and developing clearer production technologies.

## 1. Introduction

Currently, a mass of greenhouse gas emissions (GHGs), including carbon oxides, nitric oxides, and sulfur oxides, from anthropogenic economic activities due to the combustion of fossil fuels have become the leading cause of global environmental issues, such as extreme weather, drought, and rising sea levels [1,2,3]. More seriously, a part of greenhouse gas, such as sulfur oxides, not only exerts serious adverse effects on our eyes and other issues with the respiratory system [4,5], but it also has been recognized as an important source of threats to human survival [6,7]. Therefore, promoting proactive GHGs reduction has become the focus of world attention. Of particular note is that, with the increase of the population and the acceleration of industrial process in emerging economies, such as India and China, their rapid urbanization and industrialization has led to a sharp increase in the consumption of fossil fuels, with an annual growth rate of around 3% [8,9,10], and thus, it has resulted in an enormous challenge on the formulation of global environment governance policies. Consequently, for policy makers, the most important issue at this juncture is to investigate global GHGs and the relevant issues, thereby developing more effective environmental governance measure. 

In view of the above-mentioned problem, prior studies discussed the relevant issues on GHGs from multiple perspectives. Specifically, a portion of previous studies estimated the volume of GHGs for different industries, and studied emissions reduction strategies for an industry sector [11,12,13,14,15,16]. Additionally, a part of previous studies focused on GHGs in a particular region or in a group of regions [17,18,19,20,21,22,23]. Notably, considering that China was the world’s biggest emitter of GHGs, a large number of prior studies centered on measuring China’s GHGs, and investigated the corresponding reduction policies [24,25,26,27,28,29,30]. These authors primarily found that with the rapid development of China’s economy and the rapid improvement of people’s living standards, the growth in GHGs also increased sharply, resulting in serious environmental issues, such as haze and water pollution. Therefore, for stakeholders, improving energy efficiency and raising residents’ awareness of environmental protection could be feasible ways to effectively promote GHGs reduction. 

Generally, the above studies primarily focused on investigating two types of the GHGs (namely, direct and indirect emissions) and the relevant issues. The former is typically generated within the geographical boundary of a region due to fossil fuels combustion and industrial processes, whereas the latter is emitted outside the geographical boundary of a region due to energy consumption within the local region [31]. However, GHGs transferring via trade (i.e., the GHGs embodied in trade) on account of the production and consumption of products imputed to the region due to thick trade linkages are seldom analyzed. In reality, with the deepening of economic globalization, production within a region is no longer just to fulfill its own demands. Through inter-regional trade, a mass of goods, including resources and products, flow and are allocated around the world [32,33]. In other words, when inter-regional trade provided a vital mechanism to efficiently allocate products or services, it also provided a mechanism to lead to the spatial separation of the consumers and producers, and thus, the corresponding environmental impacts belonging to the consumers could be transferred to the producers due to trade relationships [34,35,36]. Thus, GHGs transferring via trade have gradually become a non-negligible source of global GHGs, accounting for around 25% of the total global GHGs since 1995 [37,38]. In particular, as economic globalization has constantly been strengthened in the past decades, global GHGs transferring via trade are on the rise at an annual growth rate of 5% [39,40,41,42]. Consequently, the impact of international trade on global GHGs has attracted attention by scholars. 

Overall, previous studies found that interregional trade could bring about the spatial transfer of GHGs, and thus, for consumers, via trade relations, they would circumvent their own GHGs reduction obligations. In other words, although developing countries have access to a great deal of resources to drive their local economic growth through trade relationships, some high-polluting and high-emission industries in many advanced economies have shifted to developing countries, and thus, environmental impacts may also be avoided by advanced economies and transferred to developing countries; the latter would also be left to shoulder more obligations for GHGs mitigation [34,39]. Consequently, some developed countries have implemented strong policies to lower GHGs, but the total volume of GHGs has continued to rise [43,44,45]. Obviously, within the context of global environmental policy, trade could lead to an unfair allocation of responsibility for reducing GHGs between the consumers and the producers, and more seriously, it is not conducive to promoting the achievement of overall GHGs reduction goals from a global perspective. 

Therefore, to assign responsibility for lessening GHGs to each region from the viewpoint of equity, for the stakeholders, conducting in-depth discussions on GHGs transferring via trade and the relevant issues, and thus, articulating balanced environmental governance measures including the imputation of GHGs reduction goals to specific regions appear to be particularly critical. In general, studies on GHGs transferring via trade and the relevant issues can be divided into two three aspects in the existing literature. 

The first aspect primarily focused on estimating one kind of GHG (i.e., carbon emissions) transferring via trade within a single region or among regions, particularly for carbon emission, and thus, analyzing the corresponding emissions mitigation strategies [36,46,47,48,49,50,51,52,53]. On the whole, of all the greenhouse gases, the total volume of carbon emissions from burning fossil fuels is larger, and it contributes 60% to the global greenhouse effect; hence, prior studies primarily focused on the related issues of carbon emissions. Nevertheless, a part of previous studies found that except for carbon emission, other greenhouse gases, such as nitric oxides and sulfur oxides, would also exert an adverse effect on ecological environmental quality [5,54]. Notably, carbon emissions contributed the most to global warming, but regarding the ability to drive global warming, other greenhouse gases, such as CH_4_, are approximately three times more than that of carbon emissions [42,55,56,57]. Thus, for policy makers, it is essential to dispose of comprehensive accounts of GHGs transferring via trade that enable stakeholders to develop more targeted reduction strategies.

The second aspect primarily centered on investigating GHGs transferring via trade and their influencing factors in a single region [24,33,38,58,59,60,61,62]. In general, given that China was the world’s biggest GHGs emitter, a mass of prior studies primarily studied the embodied GHGs in trade in China. Additionally, previous studies focused on analyzing other individual countries or regions’ GHGs embodied in trade [37,63,64]. However, with the increasing globalization of production networks and supply chains, the trade linkages between countries are becoming increasingly close in recent years, and the impacts of global trade on national GHGs accounting and the ascription of obligations for GHGs within the context of global environmental governance measures are increasingly obvious. Thus, for stakeholders, to reduce the effects of trade on imputed obligations in GHGs, a holistic analysis covering all GHGs in the world would be a precondition for formulating effective mitigation measures.

The third aspect primarily concentrated on estimating GHGs transferring via trade among a group of countries in a specific year, and thus discussed the related GHGs reduction policies [64,65,66,67] or within a certain industry [68,69,70,71,72,73,74,75]. For instance, Chen and Chen [64] investigated the GHGs released by the world economy in 2000, and found that the GHG intensities transferring via trade for the 40 sectors varied dramatically. Moreover, some researchers explored the embodied non-CO_2_ emissions, such as methane, sulfur oxide, and nitrous oxide emissions, of global major economies in a specific period [5,42,76,77,78]. Remarkably, taking the 42 major economies as an example, Han et al. [78] explored the characteristics of agricultural CH_4_ and N_2_O emissions in 2014, and found that China was the largest exporter of embodied CH_4_ and N_2_O emissions, while the US was the largest importer. 

To sum up, by exploring GHGs and the relevant issues, previous studies provided an important basis for stakeholders to understand the role of trade on regional GHGs accounting and assign obligations for GHGs reduction to each region from the perspective of being fair and reasonable within the context of environmental governance policy. However, the following main shortcomings that we are seeking to further deepen and improve also exist. 

First, few studies focused on a comprehensive analysis regarding the GHGs transferring via trade in the world, and more importantly, less emphasis was placed on the spatiotemporal evolution characteristics of the GHGs transferring via trade between regions in consecutive years. From the global environmental governance perspective, it may result in providing less effective policy implications for stakeholders to clarify the role of international trade and legitimately allocate global GHGs reduction obligations. Therefore, it is particularly of importance for us to conduct comprehensive discussions on GHGs transferring via trade at the global level for a period of time, thereby providing stakeholders with more targeted GHGs reduction policies.

Second, studies concerning influencing factors of GHGs transferring via trade had received little attention. Actually, for the direct and indirect GHGs in economic activities, a few previous studies [36,79,80] found that the growth in GHGs from the improvement of regional economic development or the increase of residents’ demand could be moderately offset by diminutions in GHGs through measures, such as lessening energy intensity or upgrading product technology, but from the GHGs transferring via trade perspective, the question is whether the above-mentioned measures are also effective and whether the regional spillover effects of these measures on the GHGs transferring via trade exist between countries. Obviously, a more comprehensive analysis could enable stakeholders to effectively develop environmental governance strategies upon a global scale. Notably, using the structural decomposition analysis within the multi-regional input-output analytical framework, although a few studies [10,81,82,83,84] explored the impacts of some determinants, such as trade openness, pollution emission intensity, the level of production technology, and requirements for final products on the carbon emissions embodied in trade changes for a group of countries. However, these studies did not take into account the spatial correlation, and the research units are treated as a single and independent individual; as a result, the possible impacts of the spatial spillover effects from influencing factors on the changes in the GHGs transferring via trade have failed to be explored. 

In fact, inter-regional economic activities are interrelated to each other, and a correlation might exist in some economic attribute values of geographically close spatial units [85]. Prior studies found that the spatial autocorrelation method could characterize the spatial relationship between a geographic unit and its adjacent units through the spatial weight matrix [86], and it is usually employed to unravel the spatial correlation of GHGs embodied in trade for a band of regions, and thus reveal their spatial distribution pattern. On this basis, the influencing factors of such spatial correlation could also be fully presented accordingly. Furthermore, previous studies verified that the changes in the level of production technology or energy structure in a region would exert non-ignorable influences on the changes in adjacent regions’ GHGs embodied in trade via production networks [87,88]. Therefore, ignoring the effects of spatial correlation may draw erroneous conclusions, thereby resulting in less useful policy recommendations for stakeholders. Additionally, it is particularly noteworthy that by using the multi-regional input-output models and econometric regression approaches, Zhong et al. [40] unraveled factors driving the embodied carbon emissions in trade changes for 39 countries from 1995 to 2011. However, these authors primarily paid more attention to analyzing the type of GHGs and the relevant issues. In reality, to develop more comprehensive environmental governance policies, a holistic analysis, including all GHGs embodied in trade, seems to be of particular importance. In addition, the discussions on the impacts of influencing factors’ spatial correlation between regions on the changes in GHGs embodied in trade have not been fully discussed. 

In this context, considering that compared to factor decomposition models, such as structural decomposition analysis, index decomposition analysis, and traditional econometric models, including least square methods, spatial econometric regression models are superior to estimating the spatial spillover effects from a set of influencing factors (i.e., explained variable) and investigating their quantitative influences on the changes in the observed explanatory variable [89,90], so these models were applied here to explore the GHGs transferring via trade and the relevant issues on the global scale over a period of time. Regrettably, however, this discussion has seldom been conducted in the existing literature. To enable stakeholders to effectively formulate more targeted environmental governance policies, this paper contributes to filling the above-mentioned gaps by conducting a comprehensive analysis on the changes in the GHGs transferring via trade for 39 major economies (all 27 EU countries and 12 major countries or regions) for a period time, and investigating the impacts of the spatial spillover effects from influencing factors on the changes in the GHGs transferring via trade on the basis of analyzing the spatial correlation between countries. 

The rest of this paper is organized as follows. The following section gives the details of the methodology and data. Section 3 and Section 4 illustrate the empirical results and the corresponding discussions, respectively. Finally, policy implications and conclusions are provided in Section 5 and Section 6, respectively. 

## 2. Methodology and Data

### 2.1. Models

#### 2.1.1. Calculation of GHGs Transferring via Trade

On the basis of the related derivations in Supplementary Material 1, the GHGs transferring via trade ($EGT_{r-s,t}$) from region r to region s for year t can be obtained as:

(1) $$EGT_{r-s,t}=\sum_{g}EGT_{r-s\left(g\right),t},$$

(2) $$EGT_{r-s\left(g\right),t}=\left[\sum_{k=1}^{N}{\left(v_{t}^{k,r\left(g\right)}\right)}^{\prime }\right]f_{t}^{r,s}+{\left(v_{t}^{r,s\left(g\right)}\right)}^{\prime }\left(\sum_{k=1}^{N}f_{t}^{s,k}\right),s\neq r,s,r,k\in N,$$

where $EGT_{r-s\left(g\right),t}$ represents the embodied g kind of GHGs in exports trade from region r to region s for year t. Moreover, for g kind of GHGs, the first half of Equation (2) is the emissions (produced from other regions) that are embodied in final goods exported by region r to other regions’ consumers (s) for year t. Actually, for region r, its final demand is provided by the rest of the world (including region r), the interregional trade in this process is bound to lead to embodied GHGs in trade. Thus, for g kind of GHGs in Equation (2), $\overset{N}{\underset{k=1}{\sum }}{\left(v_{t}^{k,r\left(g\right)}\right)}^{\prime }$ is a row vector of GHGs generated in all studied regions that are essential for one unit of an industry’s final commodities manufactured in region r for year t. $f_{t}^{r,s}$ (s,s≠r) denotes the final commodities demanded by region s, which are produced in region r for year t. Additionally, for g kind of GHGs, $v_{t}^{r,s\left(g\right)}$ (s,s≠r) is a row vector of the GHGs generated in region r that are embodied in one unit of final goods manufactured in other regions for year t. $\overset{N}{\underset{k=1}{\sum }}f_{t}^{s,k}$ represents a column vector of final commodities, manufactured in region r, are used to meet other regions’ requirements (k, k∈N). Similarly, Equations (3), (6) and (7) have the same definitions regarding these variables.

According to the above derivation, for g kind of GHGs, the total emissions including two components (namely, the demand of the economy of region r from economic activities on the local production of region r for year t and the GHGs embodied in international trade from region r to region s for year t, respectively) generated in region r ($TEP_{r\left(g\right),t}$) resulting from economic production (the total production-based GHGs for short) can be obtained as:

(3) $$TEP_{r\left(g\right),t}=\left[\sum_{k=1}^{N}{\left(v_{t}^{k,r\left(g\right)}\right)}^{\prime }\right]f_{t}^{r,r}+EGT_{r-s\left(g\right),t}.$$

Accordingly, for year t, GHGs embodied in exports ($EGE_{r,t}$) from region r to all other studied regions and GHGs embodied in imports ($EGI_{r,t}$) from all other studied regions to region r can be deduced, respectively:

(4) $$EGE_{r,t}=\sum_{g}EGE_{r\left(g\right),t},$$

(5) $$EGI_{r,t}=\sum_{g}EGI_{r\left(g\right),t},$$

(6) $$\begin{array}{c} EGE_{r\left(g\right),t}=\sum_{s\neq r}^{N}EGT_{r-s\left(g\right),t}=\left[\sum_{k=1}^{N}{\left(v_{t}^{k,r\left(g\right)}\right)}^{\prime }\right]\left(\sum_{s\neq r}^{N}f_{t}^{rs}\right)+\\ \sum_{s\neq r}^{N}\left[{\left(v_{t}^{r,s\left(g\right)}\right)}^{\prime }\left(\sum_{k=1}^{N}f_{t}^{s,k}\right)\right], \end{array}$$

(7) $$\begin{array}{c} EGI_{r\left(g\right),t}=\sum_{s\neq r}^{N}EGT_{s-r\left(g\right),t}=\sum_{s\neq r}^{N}\left[\sum_{k=1}^{N}{\left(v_{t}^{k,r\left(g\right)}\right)}^{\prime }\right]f_{t}^{sr}+\\ \sum_{s\neq r}^{N}\left[{\left(v_{t}^{r,s\left(g\right)}\right)}^{\prime }\right]\left(\sum_{k=1}^{N}f_{t}^{s,k}\right). \end{array}$$

According to the Equations (6) and (7), for a region r in year t, the total embodied GHGs in international trade ($TEGT_{r,t}$) and the balance of the embodied GHGs in international trade ($E_{r,t}^{BEGT}$) can be inferred, respectively:

(8) $$TEGT_{r,t}=EGI_{r,t}+EGE_{r,t},$$

(9) $$E_{r,t}^{BEGT}=EGE_{r,t}-EGI_{r,t}.$$

Consequently, for a region r in year t, the total GHGs generated by economic consumption ($TEC_{r,t}^{cons}$) (the total consumption-based GHGs for short) can be given as follows:

(10) $$TEC_{r,t}^{cons}=\sum_{g}TEP_{r\left(g\right),t}-E_{r,t}^{BEGT}.$$

Obviously, according to Equation (10), if $E_{r,t}^{BEGT}<0$, under a consumption-based accounting approach, the total GHGs will be aggravated for a region r in year t, which may lead to more GHGs’ reduction responsibilities to be shouldered, and vice versa.

#### 2.1.2. Econometric Regression Models and Exogenous Variables’ Selection

##### Spatial Autocorrelation Models

Through nesting spatial and temporal effects, spatial econometric regression methods could explicitly expound the impacts of influencing factors on the spatiotemporal evolution of the embodied GHGs in international trade, and thus, it is more accordant with practical circumstances [91]. Additionally, spatial econometric regression methods can provide not only the frequency of shifts in the embodied GHGs across various regions, but it also can analyze whether spatially dependence exists for the embodied GHGs in international trade between regions [92]. Therefore, spatial econometric regression methods are widely used in social and economic issues’ analysis by scholars [41,89,93]. However, prior studies thought that before conducting spatial econometric regression, the first step was to determine whether the dependent variable is spatially correlated [91,94]. In actual, spatial autocorrelation analysis could not only reveal the spatiotemporal evolution characteristics of the embodied GHGs in international trade, but it also could reflect the correlation in its spatial distribution patterns, which was a key step to correctly choose optimal models. Thus, spatial autocorrelation models, including global spatial autocorrelation (global Moran’s I) and local spatial autocorrelation (local indicator spatial autocorrelation, LISA), are usually employed here. The former suggests a general characteristic of spatial clustering of similar values among regions, by considering the spatial proximity of each value in all research units, while the latter can recognize the spatial hot spots of values among regions [91].

First, global Moran’s *I* is given as follows:

(11) $$I=\frac{n\sum_{i=1}^{n}\sum_{j=1}^{n}W_{ij}\left(x_{i}-\bar{x}\right)\left(x_{j}-\bar{x}\right)}{\sum_{i=1}^{n}\sum_{j=1}^{n}W_{ij}\sum_{i=1}^{n}(x_{j}-\bar{x})},$$

where $x_{i}$ and $x_{j}$ represents the embodied GHGs in international trade for region i and region j, respectively. $\bar{x}$ is an average of the embodied GHGs in international trade for all regions.$\;W_{ij}\;$ denotes the spatial weight matrix between region i and region j. To explore the possible impact of spatial autocorrelation among regions on the embodied GHGs in international trade, the geographic proximity factor among regions was taken into account here. Thus, in this paper, the rook matrix was adopted, that is, if one region adjacent to region i is selected with a weight of one, and otherwise, the weight is zero. n is the total number of regions. The Z value employed to test the global Moran’s I is expressed as follows:

(12) $$Z\left[I\right]=\frac{I-E\left[I\right]}{\sqrt{Var\left[I\right]}},$$

where if Z[I]>0, which indicates that it is statistically significant, and a significant positive autocorrelation exists in terms of the spatial distribution of the embodied GHGs in international trade among regions. Moreover, the larger the value (Z[I]) is, the more obvious the positive spatial correlation is, and vice versa. If Z[I]=0, it means that the spatial distribution regarding the embodied GHGs in international trade among regions is random.

Second, local spatial autocorrelation charactering the spatial association of individual units (namely, local Moran’s $I^{\prime }$) is given as follows:

(13) $$I^{\prime }=\frac{n^{2}}{\sum_{i=1}^{n}\sum_{j=1}^{n}W_{ij}}\times \frac{\left(x_{i}-\bar{x}\right)\sum_{j=1}^{n}W_{ij}\left(x_{j}-\bar{x}\right)}{\sum_{j=1}^{n}{\left(x_{j}-\bar{x}\right)}^{2}},$$

where the definitions regarding these variables in Equation (13) are the same with Equation (11). Moreover, if $I^{\prime }>0$, it indicates that for the embodied GHGs in international trade among regions, a high value is surrounded by a high value (high-high) or a low value is surrounded by a low value (low-low). If $I^{\prime }<0$, it indicates that for the embodied GHGs in international trade among regions, a low value is surrounded by a high value (low-high) or a high value is surrounded by a low value (high-low). Thus, through the Moran scatter plot, we can obtain the characteristics of spatial associations in terms of the embodied GHGs in international trade between regions, and generally, based on the positive and negative associations, four types of local spatial association between individual regions can be divided here—that is, being the positive associations, high-high type and low-low type represent high values are surrounded by high values, and low values are surrounded by low values, respectively. Summing up, these two agglomeration types indicate that a positive spatial correlation exists between two regions in terms of the embodied GHGs in international trade; in other words, a spatial agglomeration with high (or low) emissions regions exists. Being the negative associations, low-high type and high-low type denote that low values are surrounded by high values, and high values are surrounded by low values, respectively. Likewise, these two agglomeration types suggest that a negative spatial correlation exists between two regions in terms of the embodied GHGs in international trade.

##### Spatial Econometric Regression Models

As a branch of econometrics, spatial econometrics regression models are particularly specialized in dealing with spatial interaction (i.e., spatial autocorrelation) and spatial structure (i.e., spatial heterogeneity) regarding cross-sectional data model and panel data model [94]. Because the commonly used traditional econometric model, such as least square methods, usually ignored the spatial effect, it will lead to deviations in the setting of regression models accordingly, and thus bring about some incomplete estimation results. Consequently, the regression results might be a lack of explanatory power based on least square methods [95,96]. However, compared to traditional econometrics methods, the spatial correlation weight matrix is incorporated into spatial econometrics regression models covering data relevancy and heterogeneity, and thus the estimation of the corresponding results is closer to the objective facts. In the existing literature, the widely applied spatial econometric regression models consist of three types, that is, the spatial lag model, the spatial error model, and the spatial Durbin model. These three models corresponded to the different settings of spatial interaction effects, which can be expressed as follows, respectively.

(a) The spatial lag model (SLM). It primarily focused on the spatial correlation of explanatory variables among the geographical units and explored the corresponding spatial spillover effect. If spatial autocorrelation from the variable correlation of the energy structure, industrial structure, and production technology exists, the spatially lagged explanatory variable should be incorporated, which can be expressed as follows:

(14) $$y_{it}=\rho \sum_{j=1}^{n}W_{ij}y_{jt}+\beta x_{it}+u_{it}+\lambda_{it}+\varepsilon_{it},\;\varepsilon_{it}\sim IID\left(0,\rho^{2}\right),$$

where i and j denote a geographical unit; t is the time period; ρ represents the spatial autoregressive coefficient; $W_{ij}$ denotes the spatial weight matrix; β is a group of unknown parameters related to independent variables to be estimated; $u_{it}$, controlling all of the spatial fixing and the time-invariant variables, represents the spatial fixed effects; $\lambda_{it}$, controlling all of the time fixing and the space-invariant variables; and $y_{it}$ and $x_{it}$ denote the dependent variable and independent variables, respectively. Being a random variable, $\varepsilon_{it}$ is independent and identically distributed.

(b) The spatial error model (SEM). It primarily investigated the influencing degree of the variable error on the observed explanatory variables. Specifically, during the process of establishing the model, it is possible to miss some independent variables related to dependent variables that are spatially auto-correlated, and more importantly, some terms like random error may have an impact on the spatial spillover effect. For instance, the impacts from the change in independent variables of a region may spread to other regions through a space transmission mechanism, and thus these dependent variables of the region would exert a possible positive or negative influence on other regions’ dependent variable accordingly. Therefore, ignoring the spatial autocorrelation of errors will also lead to biased results, and the spatial error model can be explicitly given as follows:

(15) $$y_{it}=\beta x_{it}+u_{it}+\lambda_{it}+\varphi_{it},\;\mathrm{and}\;\varphi_{it}=\delta \sum_{j=1}^{n}W_{ij}\varphi_{it}+\varepsilon_{it},$$

where δ denotes the spatial autocorrelation coefficient, and $\varphi_{it}$ is an error item of spatial autocorrelation. The definitions regarding other variables are the same as Equation (11).

(c) The spatial Durbin model (SDM). By combing the characters of SLM and SEM, SDM could consider the spatial correlation of explanatory variables and the influencing degree of variable error on the observed explanatory variables, and thus using SDM, a better estimation effect can be obtained accordingly. Thus, one major advantage of SDM is that it not only can obtain a more unbiased and inconsistent estimation result but also the prior limitations on the extent of possible spatial spillover effects are not essentially considered [97]. The widely used SDM can be expressed as follows:

(16) $$y_{it}=\rho \sum_{j=1}^{n}W_{ij}y_{jt}+\beta x_{it}+\sum_{j=1}^{n}W_{ij}x_{i}\gamma +u_{it}+\lambda_{it}+\varepsilon_{it},\;\varepsilon_{it}\sim IID\left(0,\rho^{2}\right),$$

where  γ is the parameters associated with the spatial spillover effects among the observed explanatory variables. The definitions concerning other variables are the same as Equations (14) and (15).

##### Exogenous Variables’ Selection

In order to investigate the factors affecting the change in the embodied GHGs in international trade, there are two explanatory variables (namely, EGE and EGI, respectively) in this paper. Additionally, limited by the availability of data, two types of influencing factors (i.e., socioeconomic and technical variables), including five determinants, were selected here. Specifically, for socioeconomic variables, they contain population size, the level of regional economic development, and industrial structure. As for technical variables, they consist of the ratio between the volume of clean energy and the total energy use and energy intensity. These variables are illustrated in detail in Supplementary Material 1.2.

### 2.2. Data

The most complete data sets containing energy use, carbon emission (CO_2_), methane (CH_4_), carbon monoxide (CO), ox nitride including N_2_O and NO_X_, sulfur oxides including SO and SO_2_, nitrogen hydride (NH_3_), and non-methane volatile organic compounds (NMVOCs) at 35 sectors ((as shown in Table S1 of Supplementary Material) level for 27 EU countries and 12 major other countries or regions, plus estimates for other areas in the world from 1995 to 2011, and the GMRIO table from the WIOD were used as the raw data in this paper. Moreover, the data sets for 39 regions regarding the clean energy in a region’s share of energy consumption, energy intensity, GDP, and population size employed in our paper are from the World Bank Database, which can be free access to login to the following website: http://data.worldbank.org.cn/. In addition, to remove an influence of price factor, a part of economic data sets like GDP are *constant* prices in 1995. In addition, more detailed information concerning all explanatory variables and explained variables used in this paper are given in Table 1.

## 3. Results

### 3.1. Spatiotemporal Evolution

Overall, due to space limitations, the changes in the total greenhouse gas emissions (GHGs) transferring via trade and the total consumption-based GHGs all over the world from 1995 to 2011 are discussed in Supplementary Material Section 3. As for these, changes for the 35 sectors and 39 major countries are given here.

**Figure 1:**
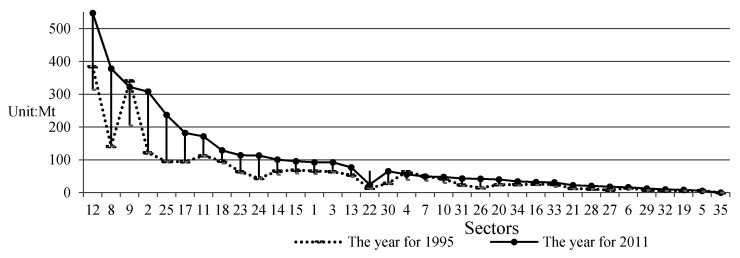
Changes of GHGs transferring via trade from 1995 to 2011 at a sector level. Notes: The maximum value and minimum value of GHGs embodied in trade in the corresponding sector between 1995 and 2011 are represented by the highest point and lowest point of the vertical line in the Figure 1, respectively. Table 1 presents the code of each sector in this study.

First, focusing on specific 35 sectors (Figure 1), sector 8, namely, coke, refined petroleum, and nuclear fuel suggested the rapidest total embodied GHGs in international trade (*TEGT*) growth rate and the greatest *TEGT* jump—to 378.29Mt in 2011, accounting for 10.69% of the total *TEGT* all over the world in 2011—an increase of more than 1.68 times compared to 1995. This was followed by sector 2 (mining and quarrying) and sector 12 (basic metals and fabricated metal). Additionally, of the 35 sectors studied, the sector with the highest *TEGT* from 1995 to 2011 is sector 12 (basic metals and fabricated metal), followed by coke, refined petroleum, and nuclear fuel, and chemicals and chemical products, respectively. Additionally, further calculation suggests that the eight with the top ranking *TEGT* values are sector 12, sector 8, sector 9, sector 2, sector 25, sector 17, sector 11, and sector 18. Similarly, compared to 1995, the share of the *TEGT* values of these eight sectors to the aggregate *TEGT* was over 55% in 2011, increased by nearly 68%. Notably, however, compared to 1995, for sector 9 and sector 22 in 2011, the former presented the minus decline, while the latter presented the positive increase.

**Figure 2:**
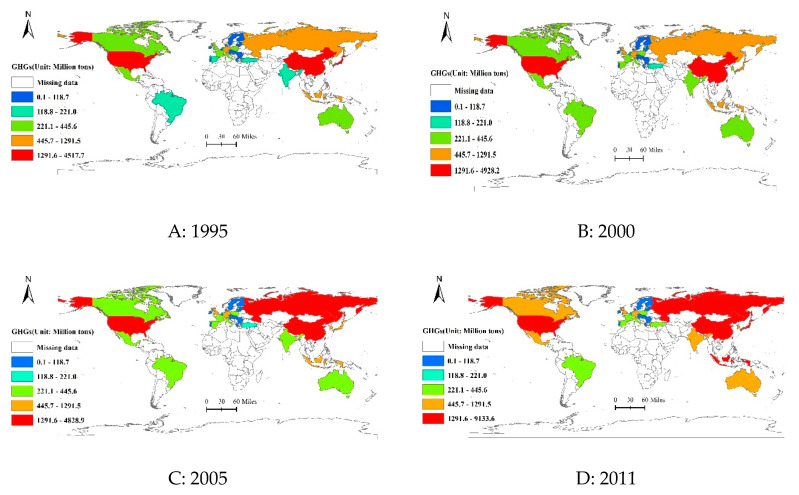
Spatiotemporal evolution characteristic of the total GHGs released by 39 major countries under a consumption-based accounting principle from 1995 to 2011.

Second, upon a national scale, to explore the impact of international trade on the assignment of GHGs reduction responsibilities, the overall spatiotemporal evolution characteristic of the total GHGs released by economic consumption for 39 major regions are displayed in Figure 2. In general, the major GHGs emitters are concentrated in China, the US, Russia, and Mexico. Thus, these regions should shoulder more responsibilities for GHGs reduction than others due to thick trade relationships under a production-based accounting principle. Moreover, further calculation indicates that the ratio between the total GHGs emitted by these four regions and the total GHGs all over the world is more than 45%, and it is on the rise, with an annual average growth rate of 5.06% during the studied period.

Notably, however, the GHGs generated by some developed economies, particularly for the European Union, remained relatively low in recent years, and these countries faced relatively little pressure to cut GHGs while allocating responsibilities. In other words, owing to close international trade relations, they circumvented their own GHGs reduction obligations from the perspective of a consumption-based accounting method, thereby alleviating the effect of the embodied GHGs. Furthermore, it should be noted that from 1995 to 2011, the GHGs volume for most regions had a tendency to increase. The region with the fastest growing GHGs is Canada, increasing to 513.14 Mt, followed by Australia and China, respectively.

**Figure 3:**
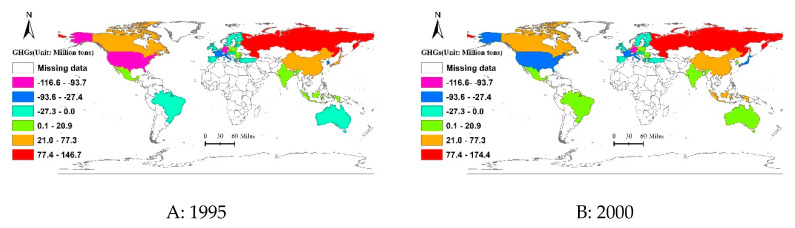

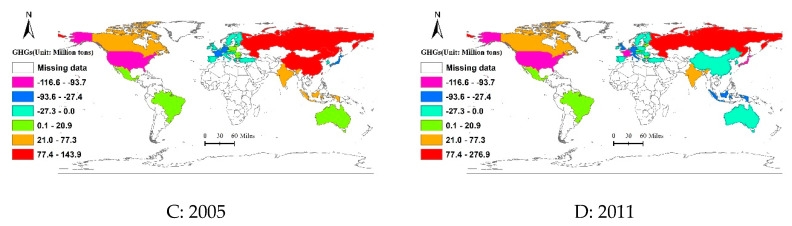
Spatiotemporal evolution characteristic of the net output of embodied GHGs in international trade from 1995 to 2011.

Last but not least, the balance of the embodied GHGs in trade for a group of 39 countries is also given in Figure 3. It should be noted that for some regions, such as China and Japan, the sign of the net output of embodied GHGs in trade changed from positive to negative from 1995 to 2011, which indicated that these countries were net emitters of GHGs, but they became net emitters as time went on. Actually, for previous studies regarding the relevant issues with the embodied carbon emissions, China was a net outflow region for a long time. However, our results show that, taking China as an example, being the world’s second largest net output of the embodied GHGs in international trade, the volume in 1995 was 77.32 Mt, while this volume was minus 9.53 Mt in 2011.

As displayed in Figure 3, regions with highest net output of embodied GHGs are primarily centered in eastern Europe and North America. Specifically, of the 39 regions studied, the region with the highest net output of embodied GHGs was Russia, and its volume maintained an increasing trend from 1995 (146.65 Mt) to 2011 (276.89 Mt) and increased by nearly two times, followed by Canada and India, respectively. While as for the net output of GHGs transferring via trade, the region with the highest net input of embodied GHGs was U.S, which increased by nearly 41% from 1995 to 2011.

With global trading relationships across regions becoming thick quickly, the embodied GHGs play a vital role in shaping national obligations for emissions mitigation in global climate policy. An issue, however, arises: What factors may influence the changes in the GHGs transferring via trade? Next, we will focus on the underlying influencing factors.

### 3.2. Influencing Factors

According to the principle of the spatial econometrics method, the basic estimation process is as follows. First of all, a spatial statistical analysis approach, such as Moran’s I, is used to test whether the dependent variable has spatial auto-correlation. Secondly, if spatial autocorrelation exists in terms of the dependent variable, and thus, based on the theoretical method of econometrics regressions, the corresponding procedures would be carried out to choose an optimal model.

#### 3.2.1. Moran’s *I* Analysis

**Figure 4:**
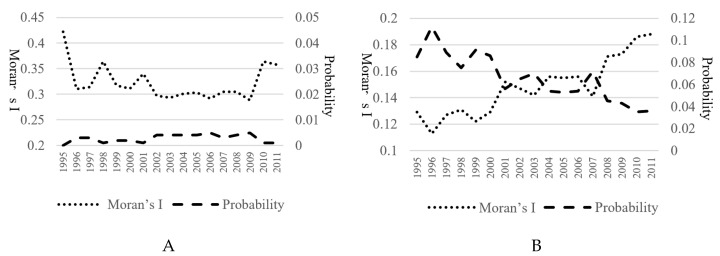
Global Moran’s *I* of the embodied GHGs in trade (**A**: The *EGE*; **B**: The *EGI*). Notes: If the value of the probability is less than 0.1, 0.05, and 0.01, which means that the normal statistics of annual Moran’s I pass under the 10%, 5%, and 1% level of the significance test.

Based on Equations (11)–(13), as shown in Figure 4, the global Moran’s I of the EGE and EGI from 1995 to 2011 was estimated, and the corresponding significance was also tested by using the normal distribution with the random permutation. First, from the EGE perspective, all of the Moran’s *I* from 1995 to 2011 were positive and the normal statistics of annual Moran’s I passed under the 5% level of significance test, which suggests that a significant spatial autocorrelation existed for the embodied GHGs in export trade at the global scale from 1995 to 2011. In other words, regions with higher or lower embodied GHGs in export trade tended to be adjacent. It is especially worth noting that as illustrated in Figure 4A, the Moran’s *I* of the EGE presented a declining tendency with a significant fluctuation from 1995 to 2009, which showed that the cluster degree of the EGE for 39 regions had a decreasing tendency. Second, looking at the EGI, as given in Figure 4B, all of the Moran’s *I* from 1995 to 2011 were positive and highly significant; more importantly, this value was on the rise, which indicates that the embodied GHGs has significant spatial autocorrelation. Moreover, its spatial agglomeration at the global level tended to be increasing significantly. On the whole, the agglomeration degree of the embodied GHGs strengthened since 1995, and regions with similar embodied GHGs tended to be agglomerated in the global spatial distribution.

Moreover, the local Moran’s *I* of the EGE and EGI from 1995 to 2011 was also estimated (due to space limitations, the maps regarding the changes of local Moran’s *I* of the EGE and EGI from 1995 to 2011 are not provided here. We will provide them on request). In general, the calculation indicates that the embodied GHGs in trade had a significant spatial agglomeration, and the regions of high-high agglomeration were distributed mainly in North America, Asia, and eastern Europe.

#### 3.2.2. Econometric Regression Results

Based on the above analysis, the embodied GHGs in international trade have significant spatial autocorrelation, which suggests that the spatial interaction between influencing factors on the embodied GHGs should not be ignored. However, traditional pooled panel regression models without spatial effects, including ordinary least squares (OLS) regression and panel regression, failed to consider spatial interactions among the influencing factors of GHGs embodied in trade, and thus, the corresponding bias regarding specification and estimated results could inevitably occur. To address this issue, by nesting spatial and temporal effects, spatial econometric regression models specialized in investigating explicitly the spatial effects of explanatory variables on the explained variables were used. Before conducting specific analysis, we should first judge whether spatial econometric regression models are superior to traditional pooled panel regression models, and then choose the optimal model based on a series of tests. To be on the safe side, four tests, including LM tests (Lagrange multiplier (hereafter, LM), Wald test, likelihood ratio (LR) test, and Hausman test, were employed to conduct a comparative analysis with the test results to select the appropriate model.

First of all, according to the related analysis in terms of the LM tests (Lagrange multiplier (hereafter, LM) test and its equivalent (i.e., robust LM test), as displayed in Table 2, for the *EGI*, LM-error, LM-lag, Robust-LM error, and Robust-LM lag were significant at the 1% level. Similarly, as for the *EGE*, although LM-error was not significant at the 10% level, other tests, such as LM-lag, Robust-LM error, and Robust-LM lag, all cannot be rejected at the 10% significance level. These test results indicate that spatially lagged dependent variable or spatially auto-correlated error term should not be ignored, and more importantly, the spatial effects of exogenous variables on the dependent variable should also be taken into account. Consequently, traditional pooled panel regression models cannot be selected here, and according to the study from Section 2.1.2, the SDM would be a more feasible option.

However, LeSage et al. [98] pointed out that SDM could be simplified to SLM or SEM under certain conditions. Therefore, compared to SLM and SEM, we need to determine that SDM is an optimal model. The relevant procedures are as follows. To determine whether the SDM can be simplified to SLM or SEM, the two hypotheses were H0: γ=0 and H0: γ+δβ=0, obeying the $\chi^{2}$ distribution with the degree of freedom, which can be identified by the Wald test and LR test. In general, according to the study by Burridge [99], if the result cannot refuse the hypothesis of H0: γ=0, and SDM can be simplified to SLM accordingly, SLM is the most appropriate model. If the result cannot refuse the hypothesis of H0: γ+δβ=0, and SDM can be simplified to SEM accordingly, SEM is the most appropriate model. If the result refuses both of the above two hypotheses, thus, SDM is the most appropriate model.

In general, as illustrated in Table 3, the result refuses both of the above two hypotheses at the 1% significance level, which indicates that SDM cannot be simplified to SLM or SEM. In other words, SDM is the best optimal model regarding analyzing the factors affecting the embodied GHGs in international trade here. In addition, to identify which specific effects model (namely, fixed effects model or random effects model) should be considered in SDM, we conducted the Hausman test accordingly. Through statistical tests, the results for the hypothesis of the random effects model of the *EGE* (97.10, 0.0000) and the *EGI* (32.11, 0.0004) both cannot be accepted at the 1% level. To conclude, we will introduce fixed effects in this study.

##### The *EGE*

In order to show the regression results more comprehensively, OLS regression (the column 2), panel regression (the column 3), random-effects (the column 4), and the SDM nesting different types of fixed effects, such as the spatial fixed (the column 5), time fixed (the column 6), and spatial and time fixed (the column 7), are given in Table 4. Based on a series of tests, particularly for the goodness of fit and the degree to which the explanatory variable meets expectations, the result indicates that we should select the SDM nesting the two-way fixed effect of space and time in the spatial regression models. On the whole, from the perspective of the regression results of the explanatory variables, the elastic coefficients of *POP*, *ES*, *EI*, *IS*, and *PGDP* are 0.213, −0.106, −0.231, −0.022, and −0.017, respectively, which indicate that except for *POP*, the other four factors have negative impacts on the *EGE* in a region. While the elastic coefficients of their spatial error are 1.134, 0.061, −0.955, 1.052, and −0.101, respectively, which indicates that the change of these factors, such as *POP*, *ES*, and *IS*, in adjacent regions have a positive influence on the *EGE*, and the change of these factors, such as *EI* and *PGDP* in neighboring regions, may drive down the *EGE*.

According to the analysis from previous studies [97,98], by estimating the coefficients of the two-way fixed effect, the spatial regression results based on the SDM (the column 7 in Table 4) fail to present the marginal effect (spillover effect) directly and calculate the direct impact of the exogenous variables on the explained variable, which may lead to some erroneous conclusions, and thus, we need to discuss the direct effect and indirect effect of these five influencing factors on the *EGE* (Table 5).

First, from the perspective of *POP*, its direct effect is found to be positive (the coefficient is 0.096) and not significant, indicating that the increase in the region’s population size has little impact on the *EGE*. One possible explanation is that the increase of the population size in a region will mainly stimulate the demand of residents or industries for products or services from other regions, and thus it also might lead to the growth of the *EGI* rather than the *EGE*. Moreover, its indirect effect is found to be positive (the coefficient is 1.047) and highly significant, which suggests that this factor has a positive spillover effect. In other words, once the indirect effect of *POP* in a region increased by 1%, the *EGE* of its adjacent regions would increase by 1.048%.

Second, looking at the *ES*, its direct effect was found to be negative (the coefficient is 0.106) and highly significant, showing that once a region’s share of clean energy increased by 1%, the corresponding *EGE* in the region would decrease by 0.106%. However, its indirect effects are positive and highly significant, which means that the proportion of the secondary sector drives up the *EGE* in neighboring regions.

Third, from the *EI* perspective, its direct effect is positive (the coefficient is 0.273) and statistically insignificant, indicating that an increase in the local energy intensity could lead to the growth of the *EGE*; however, the positive effect is not significant in 39 major countries. Furthermore, its indirect effect is found to be negative (the coefficient is 0.694) and highly significant, which suggests that this influencing factor has a negative spatial spillover effect.

Fourth, focusing on the *IS*, its direct effect is negative (the coefficient is 0.062) and insignificant, indicating that the industrial structure of the local region does not significantly reduce the *EGE*. One possible explanation is that the change of the industrial structure in a region primarily led to the change of the demand for products or services in adjacent regions rather than affecting the export of products in the region.

Last but not least, from the perspective of *PGDP*, its direct effect was found to be positive and highly insignificant. This can be explained as the change in the income level of local residents primarily exerting a significant impact on the demand of local residents for resources in neighboring regions, and has little effect on the outflow of relevant products and their corresponding GHGs. In addition, the indirect effect of the *PGDP* was found to be negative (the coefficient is 0.131) and significant, which shows that an increase in the level of region economic development drives down the *EGE* in neighboring regions.

##### The *EGI*

Similarly, as for the *EGI*, different types of econometric regression results are given in Table 6. Through conducting a comparative analysis concerning the goodness of fit and our expectations, the SDM nesting the two-way fixed effect of space and time was chosen here. Thus, as illustrated in the column 7 of Table 6, generally, we can find that *POP* and *PGDP* are positive and highly significant while *EI* have negative and significant effects on the *EGI*. As for the elastic coefficients of spatial spillover effects, *EI* and *ES* have negative spatial spillover effects, whereas other influencing factors, such as *PGDP*, *POP*, and *IS*, have positive spatial spillover effects.

Specifically, considering the robustness of regression results, the direct effect and indirect effect of these five influencing factors on the *EGI* are estimated in Table 7.

Firstly, from the *POP* perspective, it was found to have a positive (the coefficient is 1.136) and highly significant direct effect, indicating that the number of the population in a region increased by 1%, and the volume of the *EGI* increased by 1.136%. An alternative explanation is that the growth of the population size is accompanied by increasing demand for external products from neighboring regions, resulting in the *EGI*’s rise. As for its indirect effect, it is found to be negative and insignificant, indicating that this factor has no significant spatial spillover effect.

Secondly, focusing on the *ES*, its direct effect is found to be negative and insignificant, indicating that the optimization of energy structure can reduce its own *EGI*, but this effect is not significant at the global level. Actually, the above analysis found that through changing amounts of products with high pollutant emissions, the optimization of the energy structure could effectively reduce its own *EGE* rather than the *EGI* due to the little impact of the improvement in the energy structure on lessening amounts of products with high pollutant emissions from neighboring regions. As for its indirect effect, it was found to be negative and significant, which suggests that this factor has a significant negative spatial spillover effect. In other words, an increase in the ratio between clean energy and the aggregate energy use in neighboring regions drives down the *EGI*. This result is most likely because of the optimization of the energy structure, as production can be conducted more cleanly in this area, which led to reduced products with high pollutant emission factors in export trade. Consequently, while their products are imported by neighboring regions, the corresponding *EGI* would be lowered accordingly.

Thirdly, looking at the *EI*, its direct and indirect effect are both negative and highly significant (the coefficient is 0.283 and 0.251, respectively), indicating that an increase in the *EI* in a region drives down its own *EGI* and neighboring regions’ *EGI*.

Fourthly, from the *IS* perspective, its direct effect is positive but insignificant, suggesting that an increase in the proportion of the output of the secondary industry in the whole national economy drives up the *EGI*, and the effect of this factor on the region is not significant at the global level. The reason could likely be that, regarding the *EGI* primarily containing the GHGs released by the products in import trade, the industrial structure and production pattern of the region have little impact on the structure of the products imported from neighboring regions. Additionally, as for the indirect effect, it is positive and insignificant, which means that this factor has an insignificant spatial spillover effect.

Lastly, from the perspective of *PGDP*, its direct effect was found to be positive (the coefficient is 0.078) and highly significant, indicating that per capita GDP increased by 1%, and the corresponding *EGI* was raised by 0.078%. This result is most likely due to, with the improvement of regional economic development level, the income of residents increasing rapidly, which led to an increase in residents’ demand for imported products, and thus, the *EGI* will also raise accordingly. Furthermore, its indirect effect was found to be positive and insignificant, which indicates that this factor has an insignificant spatial spillover effect.

## 4. Discussions

Based on the above results, some discussions can be obtained as follows. First, from the perspective of specific 35 sectors, the energy sector, such as coke, refined petroleum, and nuclear fuel and electricity, gas and water supply, the processing manufacturing sector, such as basic metals and fabricated metal and mining and quarrying, and the material sector, such as basic metals and fabricated metal and chemicals and chemical products are the major contributors of the *TEGT*. Therefore, these sectors should shoulder greater GHGs reduction responsibilities than that of other sectors in global environmental governance measures. Additionally, focusing on a national scale, actively lessening GHGs in these four countries, such as China, the U.S., Russia, and Mexico, will have a significant positive effect on promoting global GHGs reduction.

During 1995 and 2011, our results suggests that China became a net inflow region. Moreover, a similar situation occurred in Brazil. One possible reason is that the changes in the trade structure in these regions led to the changes in embodied GHGs in international trade [40,82]. For instance, as for China in the early stage, it mainly exported labor-intensive services and products with high emissions factors, and the balance of import and export trade was relatively large, resulting in a net outflow of embodied GHGs. While with the rapid development of China’s economy, its trade structure has changed [36], that is, when the balance of import and export trade is narrowing, it is gradually importing a large number of energy-intensive products due to huge demand for domestic industry, and thus, China was a net inflow region. However, for Brazil, which is endowed with abundant natural resources, following the swift development of the global economy, large amounts of demand for its resources’ products outside the region increased dramatically, which inevitably led to a higher net output of GHGs transferring via trade.

Moreover, for regions with net output of GHGs transferring via trade, where trade provided a mechanism to efficiently assign resources, environmental impacts were also caused by thick trade relationships [35,50,100]. While for regions with net input of GHGs embodied in international trade, through trade, these countries transferred a mass of their production to other countries, and thus, a part of their responsibilities for GHGs reduction were also shifted, and the adverse impact of promoting GHGs reduction on the national economy could be avoided. Facing the influence of trade, for the perspective of environmental governance, developed economies should provide capital and technology to help these regions with net output of GHGs transferring via trade to improve the level of production technology, which is more consistent with the need for joint global emission reduction. As for regions with the net output of GHGs embodied in trade, a mass of efforts should be made to improve production technology to reduce the outflow of GHGs.

Second, according to the above analysis from Moran’s *I* analysis, the embodied GHGs at the global level have significant spatial autocorrelation, which suggests that significant spatial interaction exists. Therefore, relevant econometric models can be constructed to conduct the corresponding estimation here.

Specifically, looking at the *EGE*, from the perspective of *POP,* due to the increasing population size in a region, amounts of increasing resources produced by neighboring regions would be provided to the region’s demand accordingly, resulting in an increase in the *EGE* of neighboring regions. Focusing on the *ES*, the improvements in the region’s energy structure, particularly for the proportion of clean energy, can effectively promote more products with low emission for export, and thus, the corresponding *EGE* could lessen accordingly. Additionally, the *ES* was found to have a positive but insignificant indirect effect, which suggests that a little effect of the improvement in the local energy structure on the *EGE* of neighboring regions exists here. An alternative reason is that the optimization of the energy structure in a region is difficult to enhance high-technology products with low emission or reduce the amount of carbon in the exported trade in neighboring regions. From the *EI* perspective, an increase in the *EI* in neighboring regions drives down the *EGE*. The reason might be explained that owing to the increase of the local *EI*, on the one hand, the technical level of production in this area was improved accordingly, resulting in lowered demand for energy-intensive products from surrounding regions. On the other hand, the improvement of production technology in a region, driven by the inter-regional knowledge spillover effect, may bring about the improvement of production technology in neighboring regions. In this context, it would produce more non-energy-intensive products with high emission factors than before, and thus, the *EGE* from neighboring regions would reduce accordingly. Looking at the *IS*, due to the continuous optimization of the industrial structure in the region, the energy-intensive industry with high pollutant factors that need to meet the demand of domestic production and consumers would be transferred to neighboring areas to some extent [82], and thus, the GHGs are also shifted accordingly. Consequently, the *EGE* would be driven up. Additionally, from the perspective of *PGDP*, an increase in the level of regional economic development of neighboring regions would lead to an increase of residents’ awareness of environmental protection, resulting in lowered need for energy-intensive products from neighboring regions. Consequently, the *EGE* would be reduced accordingly.

Furthermore, looking at the *EGI*, from the *POP* perspective, by stimulating the increasing demand for products from neighboring regions, the growth of the population size in the region primarily drives up its own *EGI* rather than the *EGI* of neighboring regions. Focusing on the *ES*, for the related stakeholders, the above analysis suggests that although increasing the proportion of clean energy cannot effectively mitigate the *EGI*, it can significantly lessen the *EGI* of neighboring regions, thus improving the energy mix is conducive to promoting global GHGs reduction. Looking at the *EI*, the increase of the local energy intensity accompanies the improvement in regional production technology, which could effectively enhance energy efficiency and reduce the demand for energy-intensive products [36,40]. From the *IS* perspective, optimizing the industrial structure in neighboring regions denotes a reduction in the proportion of the output of the secondary industry in the whole national economy, and thus, parts of production activities originally needed to meet its own demands might be shifted to other regions. Consequently, the volume of exports and the corresponding *EGE* would increase, while the volume of imports and the corresponding *EGI* in neighboring regions might rise to some extent accordingly. From the perspective of *PGDP*, the change in the population size primarily has a positive impact on the changes of its own residents’ demand, and it is difficult to affect the demand of residents in neighboring regions.

## 5. Policy Implications

First, according to the above results, it transpires that, with the increase in the total GHGs embodied in trade, for developed economies with net input of GHGs embodied in international trade, the impacts of the implementation of strict GHGs reduction policies on improving environmental quality are not significant, and thus, it is urgent for policy makers to discuss reduction strategies from the global perspective. Additionally, to curtail the impact of trade, developed economies should provide capital and technology to help these regions with net output of GHGs embodied in trade to improve their production technology, and transform from a resource-driven economy that excessively relies on traditional fossil energy into a technology-driven economy characterized by low carbon and green economy industry development. For regions with net output of GHGs transferring via trade, while promoting economic development, efforts should be made to improve energy efficiency or introduce advanced energy technology so as to lessen the outflow of GHGs. With global trading relationships becoming increasingly close, the embodied GHGs in trade are playing a crucial role in shaping national obligations for GHGs mitigation in global environmental governance measures. Therefore, for the stakeholders, it can be concluded that GHGs transferring via trade should be explicitly accounted for in sound environmental policy design but also that the GHGs assessment should be conducted at multiple spatial scales to unravel the complexity of trade relationships.

Second, considering that the changes in population size exert a significant impact on the changes in GHGs transferring via trade, thus how to effectively lower the large increase in resources consumption resulting from the increase in population size is particularly critical. Additionally, our results indicate that with the improvement of the level of regional economic development, the rise of current residents’ living standards will promote the growth of GHGs transferring via trade, and this factor has significant spatial spillover effects. Therefore, these results may imply that it is difficult for the stakeholders to achieve global GHGs reduction targets by relying on some regions, such as China and EU, to reduce emissions aggressively, which may require cross-region collaboration. In other words, as other countries, such as Australia, Russia, and Canada, are developed economies with abundant fossil fuel, from the perspective of environmental governance, it is particularly important to vigorously develop clean energy technologies. Moreover, for emerging economies, such as Brazil and India, endowed with massive domestic demand resulting from rapid industrialization, according to the study by Cox and Collins et al. [101] and Jiang and He et al. [102], an environmental Kuznets curve exists between the level of regional economic development and GHGs, which is with the continuous improvement of level of regional economic development, particularly for residents’ income level, their awareness of environmental protection will be enhanced, which is conducive to promoting energy conservation and emissions reduction as well as the improvement of regional environmental quality. Therefore, for policy makers, improving residents’ awareness of environmental protection and guiding people’s green consumption behavior would be an effective way to promote global GHGs reduction.

Lastly, as globalization continued to deepen, the geographical separation of economic activities among regions became more apparent, resulting in a phenomena that the optimization of the regional industrial structure has no obvious facilitating effect on both the emission reduction of GHGs in a region and the overall GHGs reduction of the world. Thus, for the stakeholders, a mass of efforts to promote industrial restructuring and upgrading to mitigate the impact of trade may need to be treated with a caution. However, the energy utilization efficiency and energy structure can exert positive impacts on lowering the impacts of embodied GHGs, and more importantly, these factors have significant spatial spillover effects. In other words, within the context of global environmental governance policy, for policy makers, promoting GHGs reduction cooperation in sharing of energy technologies and developing clearer production technologies could reduce the effects of trade on the accounting of regional GHGs and the assignment of obligation for GHGs reduction, and it is also conductive to promoting actively global participation in GHGs reduction.

## 6. Conclusions

The aim of this paper was to promote a more systematic and comprehensive understanding of GHGs embodied in international trade at the global level. To this end, on the basis of investigating the spatiotemporal evolution of GHGs transferring via trade for 39 major countries from 1995 to 2011 using the multi-regional input-output analysis models, we employed spatial econometric regression approaches to analyze and discuss the corresponding influencing factors. Moreover, we articulated more targeted global environmental governance measures. The main conclusions are as follows.

First, for the spatiotemporal evolution, overall, the total GHGs transferring via trade surged sharply, indicating that trade is boosting a spatial displacement of the producers and the consumers. Furthermore, coke, refined petroleum, and nuclear fuel exhibited the rapidest *TEGT* growth rate and the greatest *TEGT* jump, accounting for 10.69% of the total *TEGT* in the world in 2011. Additionally, the total consumption-based GHGs presented a slow rising trend, increasing by 43.58% in 2011. More importantly, the ratio between the total GHGs transferring via trade and the total global consumption-based GHGs was approximately 7% and is on the rise. Additionally, looking at the national scale, the major GHGs emitters are concentrated in China, US, Russia, and Mexico.

Second, as for influencing factors, a significant spatial autocorrelation exists on a global scale, indicating that spatial interaction among the influencing factors on embodied GHGs in trade also exists. Specifically, for the *EGE*, some influencing factors, such as *EI* and *PGDP*, have negative spillover effects, which means that for a region, the change of these factors in neighboring regions could drive down its own *EGE*, while other influencing factors, such as *POP* and *IS*, have positive impacts on the *EGE* in neighboring regions. As far as the *EGI* is concerned, the influencing factors, such as *ES* and *EI*, have significant negative spatial spillover effects. In other words, for a region, a rise in the proportion of clean energy or an improvement in the energy intensity in neighboring regions could drive down its own *EGI*, while for the rest of the influencing factors, such as the direct effect of *POP* and *PGDP*, will be positive and highly significant.

Lastly, to reduce the impact of international trade, developed economies should provide capital and technology to help these regions with the net output of GHGs embodied in international trade. In addition, for policy makers, facilitating GHGs reduction cooperation among regions in terms of the sharing of energy technologies and developing clearer production technologies could reduce the effect of international trade on the accounting of regional GHGs and the assignment of obligation for GHGs reduction, and it is also conductive to promoting global participation in GHGs reduction.

## Figures and Tables

**Table 1:** Descriptive statistics of the data set involved in this paper.

Variable	Interpretation	Unit	Mean	S.D.	Minimum	Maximum
*ln* EGI	GHGs embodied in imports trade	KG tons	16.58	1.38	13.01	19.49
*ln* EGE	GHGs embodied in exports trade	KG tons	16.46	1.66	11.14	19.48
*lnPOP*	Population size	Million	16.77	1.91	12.82	21.02
*lnPGDP*	The level of regional economic development	Dollar	8.92	1.69	3.84	11.36
*lnEI*	Energy intensity	dollar/KG	−2.04	0.36	−2.76	−1.07
*lnES*	The clean energy in a region’s share of energy consumption	%	1.63	1.95	−9.21	3.93
*lnIS*	The share of the secondary sector in the whole national economy	%	−0.87	0.23	−1.96	−0.38

**Table 2:** Test results of LM for the *EGE* and *EGI*.

Variable	LM-Error	LM-Lag	Robust-LM Error	Robust-LM Lag
The *EGI*	54.725 ***	86.331 ***	30.173 ***	61.779 ***
The *EGE*	0.137	3.034 *	2.761 **	5.658 **

Notes: t statistics in parenthesis. * *p* < 0.10, ** *p* < 0.05, *** *p* < 0.01.

**Table 3:** Test results of the Wald test and LR test for the *EGE* and *EGI*.

Variable	LR-Lag	Wald-Lag	LR-Error	Wald-Error
The *EGE*	12.943 **	12.139 **	12.028 **	11.986 **
The *EGI*	25.271 **	24.008 **	25.339 **	23.846 **

Notes: t statistics in parenthesis. * *p* < 0.10, ** *p* < 0.05, *** *p* < 0.01.

**Table 4:** The econometric regression results concerning the embodied GHGs in export trade.

Variable	OLS Regression	Panel Regression	SDM			
			Random-Effects	Spatial Fixed-Effects	Time Fixed-Effects	Spatial and Time Fixed-Effects
*LnPOP*	0.336 (0.93)	0.208 *** (1.27)	0.171 (1.02)	0.484 (1.41)	0.676 * (1.76)	0.213 (1.01)
*LnES*	0.149 *** (4.55)	−0.091 *** (−2.33)	−0.093 *** (−2.82)	−0.100 *** (−5.10)	0.084 ** (2.49)	−0.106 *** (−7.35)
*LnEI*	0.519 *** (2.95)	−0.507 *** (−2.66)	0.164 (0.66)	0.103 (0.63)	1.600 *** (8.03)	−0.231 (−1.88)
*LnIS*	2.431 *** (9.37)	0.096 (0.29)	0.088 (0.26)	0.005 (0.02)	2.090 *** (8.29)	−0.022 (−3.46)
*LnPGDP*	0.108 *** (2.78)	0.034 (0.65)	0.025 (0.74)	0.012 (0.29)	0.224 *** (5.46)	−0.017 (−2.24)
*Constant*	4.507 *** (8.77)	0.920 (1.10)	1.560 (1.63)	-	-	-
W*LnPOP	-	-	0.151 (0.68)	1.352 ** (2.52)	−0.027 (−0.49)	1.134 ** (1.37)
W*LnES	-	-	0.034 (0.45)	0.047 (0.67)	0.284 *** (3.93)	0.061 (0.01)
W*LnEI	-	-	−1.155 *** (−3.32)	−1.054 *** (−4.72)	−2.288 *** (−9.32)	−0.955 *** (1.61)
W*LnIS	-	-	0.757 ** (2.05)	1.012 *** (2.72)	0.160 (0.40)	1.052 ** (−1.20)
W*LnPGDP	-	-	−0.127 (−1.36)	−0.147 ** (−2.55)	−0.498 *** (−8.11)	−0.101 ** (1.62)
Corrected R^2^	0.1978	0.0112	0.5735	0.1363	0.3460	0.9637
Number of obs	663	663	663	663	663	663

Notes: t statistics in parenthesis. * *p* < 0.10, ** *p* < 0.05, *** *p* < 0.01.

**Table 5:** Direct, indirect, and total effects for the *EGE*.

Variable	Direct Effect	T Statistics	Indirect Effect	T Statistics	Total Effect	T Statistics
*LnPOP*	0.096	0.21	1.047 **	2.41	1.143 **	2.39
*LnES*	−0.106 ***	−5.55	0.053	0.86	−0.053	−0.78
*LnEI*	0.273	1.62	−0.694 ***	−3.33	−0.420 *	−1.85
*LnIS*	−0.062	−0.27	0.727 **	2.11	0.664	1.43
*LnPGDP*	0.007	0.16	−0.131 **	−2.38	−0.124 **	−1.99

Notes: * *p* < 0.10, ** *p* < 0.05, *** *p* < 0.01.

**Table 6:** The econometric regression results concerning the embodied GHGs in import trade.

Variable	OLS Regression	Panel Regression	SDM			
			Random-Effects	Spatial Fixed-Effects	Time Fixed-Effects	Spatial And Time Fixed-Effects
*LnPOP*	0.649 *** (51.98)	0.649 *** (8.59)	0.707 *** (8.13)	1.429 *** (7.49)	0.751 *** (63.74)	0.969 *** (3.77)
*LnES*	0.108 *** (9.51)	0.014 (0.82)	0.006 (0.33)	−0.004 (−0.34)	0.035 *** (2.84)	−0.014 (−1.18)
*LnEI*	−0.033 (−0.54)	−0.524 *** (−3.47)	−0.369 ** (−2.09)	−0.395 *** (−4.28)	0.368 *** (5.28)	−0.275 *** (−2.65)
*LnIS*	−0.210 *** (−2.34)	−0.057 (−0.16)	0.034 (0.10)	0.081 (0.67)	0.066 (0.88)	0.079 (0.57)
*LnPGDP*	0.468 *** (35.01)	0.152 ** (2.32)	0.095 (1.25)	0.078 *** (3.41)	0.448 *** (32.03)	0.077 *** (3.04)
*Constant*	−3.756 *** −21.15)	−1.649 ** (−2.65)	−1.226 ** (−2.00)	-	-	-
W*LnPOP	-	-	−0.224 (−1.46)	−0.268 (−0.86)	−0.310 *** (−6.64)	0.323 (1.02)
W*LnES	-	-	−0.136 ** (−2.24)	−0.148 *** (−3.80)	0.138 *** (4.74)	−0.137 *** (−3.31)
W*LnEI	-	-	−0.310 (−1.02)	−0.374 ** (−2.98)	−0.207 (−1.48)	−0.333 ** (−2.30)
W*LnIS	-	-	0.044 (0.12)	0.315 (1.50)	−0.617 *** (−3.73)	0.480 * (1.95)
W*LnPGDP	-	-	0.027 (0.52)	−0.021 (−0.63)	0.032 (0.76)	0.057 (1.47)
Corrected R^2^	0.8628	0.2745	0.7154	0.5953	0.8636	0.9839
Number of obs	663	663	663	663	663	663

Notes: t statistics in parenthesis. * *p* < 0.10, ** *p* < 0.05, *** *p* < 0.01.

**Table 7:** Direct, indirect and total effects for the *EGI*.

Variable	Direct Effect	T Statistics	Indirect Effect	T Statistics	Total Effect	T Statistics
*LnPOP*	1.136 ***	4.64	−0.075	−0.31	1.061 ***	3.77
*LnES*	−0.010	−0.94	−0.120 ***	−3.30	−0.130 ***	−3.18
*LnEI*	−0.283 ***	−3.08	−0.251 **	−2.09	−0.534 ***	−3.92
*LnIS*	0.031	0.24	0.187	0.92	0.218	0.78
*LnPGDP*	0.078 ***	3.36	0.002	0.07	0.080 **	2.12

Notes: * *p* < 0.10, ** *p* < 0.05, *** *p* < 0.01.

## Supplementary Materials

The following are available online at https://www.mdpi.com/1660-4601/17/14/5065/s1, Supplementary Materials: Spatiotemporal evolution of global greenhouse gas emissions transferring via trade: Influencing factors and policy implications.
