# Supplementary material for: Spatiotemporal Evolution of Global Greenhouse Gas Emissions Transferring via Trade: Influencing Factors and Policy Implications
Source: Int J Environ Res Public Health. 2020 Jul 14;17(14):5065. doi: 10.3390/ijerph17145065 (PMC7400448; doi:10.3390/ijerph17145065)
Supplement: Supplementary file 1 [file ijerph-17-05065-s001.pdf]

Supplemental section: Spatiotemporal evolution of global greenhouse gas emissions transferring via trade: Influencing factors and policy implications

1Models

1.1Calculation of GHGs transferring via trade

Based on the global multi-regional input-output (GMRIO) table from the world input-output database (WIOD), and considering an equilibrium relationship between the columns and the rows in input-output analysis theory, the intermediate deliveries can be defined as follows:

$$Z_t = \begin{bmatrix} Z_t^{1,1} & \cdots & Z_t^{1,n} & \cdots & Z_t^{1,N} \\ \vdots & \ddots & \vdots & \ddots & \vdots \\ Z_t^{n,1} & \cdots & Z_t^{n,n} & \cdots & Z_t^{n,N} \\ \vdots & \ddots & \vdots & \ddots & \vdots \\ Z_t^{N,1} & \cdots & Z_t^{N,n} & \cdots & Z_t^{N,N} \end{bmatrix} \quad (1)$$

Where $Z_t^{r,s}$ represents intermediate input from region r to region s for year t. Z_t is the corresponding matrix with intermediate deliveries. Furthermore, as if $z_{i,j,t}^{r,s}$ denotes intermediate input from sector i in region s to sector j in region r, and thus, for year t, the intermediate input coefficients could be deduced as $a_{ij,t}^{rs} = z_{ij,t}^{rs}/y_{j,t}^{s}$, and $y_{j,t}^{s}$ denotes the output of sector j in region s, $r,s,n \in N, i,j \in J, N = 39, J = 35$. Additionally, for the convenience of calculation, an intermediate input matrix (A_t) with a similar structure is established here, which reflects the national production technology.

$$A_t = \begin{bmatrix} A_t^{1,1} & \cdots & A_t^{1,n} & \cdots & A_t^{1,N} \\ \vdots & \ddots & \vdots & \ddots & \vdots \\ A_t^{n,1} & \cdots & A_t^{n,n} & \cdots & A_t^{n,N} \\ \vdots & \ddots & \vdots & \ddots & \vdots \\ A_t^{N,1} & \cdots & A_t^{N,n} & \cdots & A_t^{N,N} \end{bmatrix} \quad (2)$$

Similarly, $A_t^{r,s}$ represents intermediate input from region r to region s for year t. Also, based on the GMRIO table, the final demand matrix (F_t) can be defined as follows:

$$F_t = \begin{bmatrix} f_t^{1,1} & \cdots & f_t^{1,n} & \cdots & f_t^{1,N} \\ \vdots & \ddots & \vdots & \ddots & \vdots \\ f_t^{n,1} & \cdots & f_t^{n,n} & \cdots & f_t^{n,N} \\ \vdots & \ddots & \vdots & \ddots & \vdots \\ f_t^{N,1} & \cdots & f_t^{N,n} & \cdots & f_t^{N,N} \end{bmatrix} \quad (3)$$

Where $f_t^{r,s}$ represents the final demand from region r to region s for year t. F_t is the corresponding matrix with final demand. Moreover, according to an equilibrium relationship between the columns and the rows in input-output analysis theory, we can obtain:

$$x_t^r = \sum_{s=1}^{N} Z_t^{rs} u + \sum_{s=1}^{N} f_t^{rs} \quad (4)$$

Where x_t^r is the aggregate output of region r for year t. u is a vector matrix composed of ones with a similar structure as the intermediate demand coefficient matrix ($Z_t^{r,s}$). According to the aggregate output including intermediate input and final demand in an output-input analysis

framework, equation (4) can be rewritten as $x_t = Z_t u + F_t u$. Also, based on the equations (1)~(3), we can also obtain: $x_t = A_t x_t + F_t u$. Based on the above derivation, the following equations accordingly can be obtain: $Z_t u = A_t x_t$, $x_t = (I - A_t)^{-1} F_t u$, and $M_t \equiv (I - A_t)^{-1}$, where M_t and I are Leontief inverse matrix and unit matrix, respectively. Consequently, the full emission coefficient matrix can be defined as follows:

$$V_t = \begin{bmatrix} (w_t^1)' M_t^{1,1} & \cdots & (w_t^1)' M_t^{1,n} & \cdots & (w_t^1)' M_t^{1,N} \\ \vdots & \ddots & \vdots & \ddots & \vdots \\ (w_t^n)' M_t^{n,1} & \cdots & (w_t^n)' M_t^{n,n} & \cdots & (w_t^n)' M_t^{n,N} \\ \vdots & \ddots & \vdots & \ddots & \vdots \\ (w_t^N)' M_t^{N,1} & \cdots & (w_t^N)' M_t^{N,n} & \cdots & (w_t^N)' M_t^{N,N} \end{bmatrix} \quad (5)$$

Where $M_t^{r,s}$ denotes Leontief inverse matrix of final demand from region r to region s for year t. Of all GHGs, for g kind of GHGs including eight emissions such as CO_2, CH_4, CO, N_2O, NO_X, SO_2, NH_3, and NMVOC, respectively, its emissions' coefficients for sector i in region r is expressed as: $w_{i,t}^{r(g)} = e_{i,t}^{r(g)} / x_{i,t}^r$, where $x_{i,t}^r$ the output of sector i in region r for year t. $w_{i,t}^{r(g)}$ denotes the volume of g kind of GHGs in sector i of per unit of the aggregate output in region r for year t (Unit: t/ ten thousand dollars). Also, according to equation (5), we can obtain: for g kind of GHGs, $(v_t^{r,s(g)})' = (w_t^{r(g)})' M_t^{rs}$, represents the GHGs released in region r for one unit of final demand in region s produced in region r for year t.

1.2Exogenous variables' selection

1) Population size (*POP*). previous studies (Merino et al., 2012; Habiyaremye, 2016; Simon, 2019) have shown that the growth in the population size in a region will stimulate the demand for products or services inside and outside the region, which will lead to the rise of GHGs to a certain extent, and thus, it has gradually become one of the main factors deteriorating the global ecological environment. Investigating its reason, on one hand, the continuous increase of population will directly lead to the increase of the demand for various resources, and thus, the influence of human economic activities on the regional ecological environment will become more and more prominent (Galli, 2015), which will impose great pressure on the environment. On the other hand, as for GHGs, the demand for energy products outside the region from increasing population is also increasing, and thus, it also brings about reassigning the production of products abroad or importing replacement (Garmendia et al., 2016; Da Silva et al., 2017). Consequently, the trend in the interregional transfer of the GHGs resulted from international trade is accelerating accordingly. Thus, an increase in regional population size may promote the flow of the GHGs. Hence, this paper hypothesizes that population size exerts a positive effect on GHGs embodied in export and import trade here. The number of population for each country or region is employed to represent the population size variable in this study.

2) The level of regional economic development (*PGDP*). A few studies found that, particularly for amounts of developing countries such as India and China, with the continuous improvement of regional economic development level, regional GHGs are also increasing at a faster pace, resulting in the deterioration of ecological environment and environmental pollution (Mi et al., 2016; Brizga et al., 2017). Looking at its reason, the growth of regional economy stimulates the demand for energy-intensive products with high emission factors (Bagheri et al., 2018; Springer et al., 2019),

and thus, the continued increase in global fossil energy consumption might lead to a surge in global energy trade, which caused a successive rise in the embodied GHGs in international trade accordingly. Actually, previous studies have shown that some pollutants like carbon emissions and sulfur oxide flow from developing economies into developed economies distinguished by high-income-level. Hence, it is hypothesized that the level of regional economic development might have a positive impact on GHGs transferring via import trade, while it may drive down GHGs transferring via export trade. Per capita gross demotic product (GDP) is used here to denote the level of regional economic development.

3) Energy intensity (*EI*). Being a wildly-used index to quantity the efficiency of energy use, energy intensity primarily can reflect the technical level of energy saving and emission reduction in a region, which exert an important effect on lessening GHGs (Feng et al., 2015; Zhang et al., 2016). Actually, based on the model derivation in section 2.1.1, through affecting the direct emission coefficient, energy intensity also plays a vital role in GHGs transferring via trade. Additionally, a part of prior studies found that the higher energy efficiency in a region, the lower the embodied pollutant emissions in export trade, whereas the impact of energy efficiency on the embodied pollutant emissions in import trade is relatively weak. In this study, we hypothesize that energy efficiency may have a negative effect on GHGs transferring via import and export trade, and the volume of GDP per unit of energy consumption in a region is employed to represent energy intensity.

4) The clean energy in a region's share of energy consumption (*ES*). Previous studies have shown that clean energy technologies can improve regional ecological environmental quality (Wang et al., 2019). Investigating its reason, this is mainly due to the fact that the fossil fuel energy structure dominated by coal, oil and natural gas will inevitably drive up GHGs transferring via trade in the production process (Vandani et al., 2016). Therefore, enhancing the proportion of regional clean energy in national economic production can produce more products with low emission, which thus can effectively lower products with high emission factors in export trade. For this purpose, optimizing regional energy structure or adjusting the proportion of clean energy such as water energy, nuclear energy, geothermal energy and solar energy in regional production structure, which may drive significantly down GHGs transferring via import and export trade. Hence, it is hypothesized that the proportion of clean energy can drive down GHGs transferring via trade in this paper.

5) Industrial structure (*IS*). Being the largest fossil-fuels consumer, the secondary sector emits the most GHGs (Yang et al., 2016; Liu et al., 2017). Especially for some emerging economies, such as China and India, regional industrial development is in a period of rapid development. In the structure of national economy, the secondary sector dominated by energy and mining industry has a large demand for fossil energy, and it is also an important source for the production of energy-intensive products, which has become one of the significant factors to promote the spatial transfer of GHGs. Therefore, in the global trade structure, the level of industrialization has a significant and positive impact on GHGs transferring via trade (Zhong et al., 2018; Zhong et al., 2019). Thus, we hypothesized that the proportion of the output of the secondary industry in the total output may drive up on GHGs transferring via trade, and the share of the secondary sector is used to represent here.

2Data

The WIOD is used as the original data for our GMRIO table including 35 sectors are given in Table 1.

Table 1 Sector classification for global multi-regional input-output table

Sector	Code	Sector	Code
Agriculture, Hunting, Forestry and Fishing	1	Construction	18
Mining and Quarrying	2	Sale, Maintenance and Repair of Motor Vehicles and Motorcycles; Retail Sale of Fuel	19
Food, Beverages and Tobacco	3	Wholesale Trade and Commission Trade, Except of Motor Vehicles and Motorcycles	20
Textiles and Textile Products	4	Retail Trade, Except of Motor Vehicles and Motorcycles; Repair of Household Goods	21
Leather, Leather and Footwear	5	Hotels and Restaurants	22
Wood and Products of Wood and Cork	6	Inland Transport	23
Pulp, Paper, Paper , Printing and Publishing	7	Water Transport	24
Coke, Refined Petroleum and Nuclear Fuel	8	Air Transport	25
Chemicals and Chemical Products	9	Other Supporting and Auxiliary Transport Activities; Activities of Travel Agencies	26
Rubber and Plastics	10	Post and Telecommunications	27
Other Non-Metallic Mineral	11	Financial Intermediation	28
Basic Metals and Fabricated Metal	12	Real Estate Activities	29
Machinery, Nec	13	Renting of M&Eq and Other Business Activities	30
Electrical and Optical Equipment	14	Public Admin and Defence; Compulsory Social Security	31
Transport Equipment	15	Education	32
Manufacturing, Nec; Recycling	16	Health and Social Work	33
Electricity, Gas and Water Supply	17	Other Community, Social and Personal Services	34
		Private Households with Employed Persons	35

3Spatiotemporal evolution

Based on equation (8) and (10) in the body, as displayed in Fig. 1, generally, the total GHGs transferring via trade and the total consumption-based GHGs all over the world are estimated here. Specifically, for the former, it is on the rise from 1995 (1.26Gt) to 2011 (1.99Gt), with an annual growth rate of approximately 3.34%, which indicates that with the deepening of economic globalization, international trade is accelerating the geographic separation of the producers and the consumers, and the corresponding adverse environmental impacts on economic development and air quality belonged to the consumers could be transferred to the GHGs emitted during the production of consumable items due to thick international trade relationships. In this context, the impacts of the implementation of strict regional GHGs reduction policies in some regions on improving global environmental quality are not significant, and thus, it is urgent for the policy-makers to discuss GHGs reduction strategies from the global perspective. As for the latter, compared to the year for 1995 (18.12Gt), the total consumption-based GHGs are on a slow rise, increased by 43.58% in 2011 (26.01Gt), which suggests that the mission for promoting global GHGs mitigation is still a work in progress. To cope with global environmental pollutant emissions, how to actively promote balanced environmental governance appears to be particularly critical. Moreover, further calculation shows that the share of the total GHGs transferring via trade to the total consumption-based GHGs all over the world is nearly 7% and presents a gradual upward trend[1], which indicates

[1] It should be noted that, according to the studies by Zhong and Jiang et al. (2018) and Zhong and Zhang et al.

that the GHGs transferring via trade has an important and far-reaching impact on the formulation of global environmental governance policy.

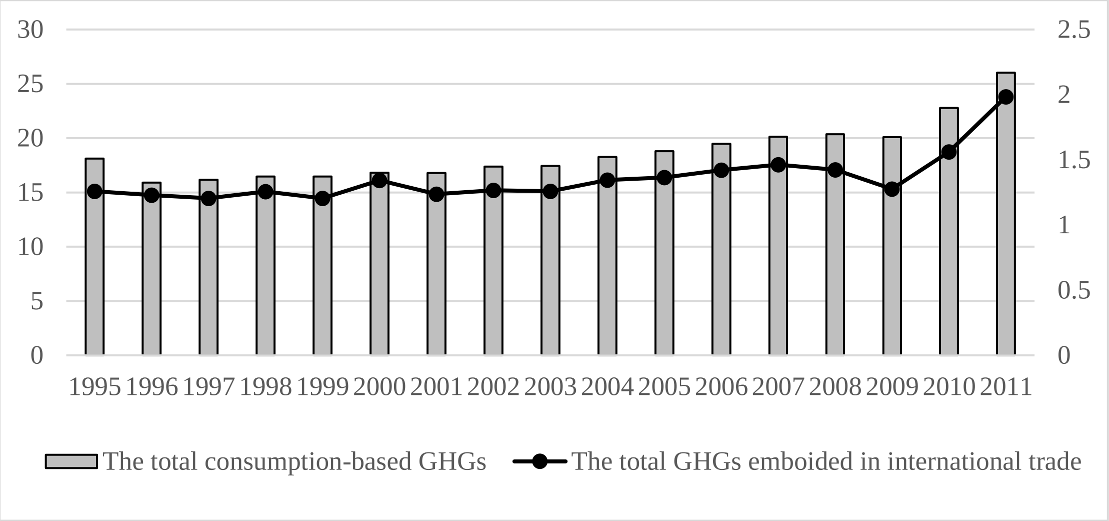

Note: The left side of the axis represents the total consumption-based GHGs (Unit: Gt). The right side of the axis represents the total GHGs embodied in international trade including the EGE and the EGI. (Unit: Gt).

Fig.1. Changes in the total consumption-based GHGs and the GHGs transferring via trade between 1995 and 2011

References

Bagheri M, Guevara Z, Alikarami M, et al. Green growth planning: A multi-factor energy input-output analysis of the Canadian economy[J]. Energy Economics, 2018, 74: 708-720.

Brizga J, Feng K, Hubacek K. Household carbon footprints in the Baltic States: A global multi-regional input–output analysis from 1995 to 2011[J]. Applied Energy, 2017, 189: 780-788.

Da Silva R F B, Batistella M, Moran E F. Socioeconomic changes and environmental policies as dimensions of regional land transitions in the Atlantic Forest, Brazil[J]. Environmental Science & Policy, 2017, 74: 14-22.

Feng K, Davis S J, Sun L, et al. Drivers of the US CO_2 emissions 1997–2013[J]. Nature Communications, 2015, 6: 7714.

Habiyaremye A. Is Sino-African trade exacerbating resource dependence in Africa?[J]. Structural Change and Economic Dynamics, 2016, 37: 1-12.

Liu Z, Adams M, Cote R P, et al. Comprehensive development of industrial symbiosis for the response of greenhouse gases emission mitigation: Challenges and opportunities in China[J]. Energy Policy, 2017, 102: 88-95.

Merino G, Barange M, Blanchard J L, et al. Can marine fisheries and aquaculture meet fish demand from a growing human population in a changing climate?[J]. Global Environmental Change, 2012, 22(4): 795-806.

Mi Z, Zhang Y, Guan D, et al. Consumption-based emission accounting for Chinese cities[J].

(2019), the ratio between the volume of the total embodied carbon emissions (sulfur oxides) in international trade and the total volume of global total consumption-based carbon emissions (sulfur oxides) is 26%(10%).

Applied Energy, 2016, 184: 1073-1081.

Galli A. On the rationale and policy usefulness of Ecological Footprint Accounting: The case of Morocco[J]. Environmental Science & Policy, 2015, 48: 210-224.

Garmendia E, Urkidi L, Arto I, et al. Tracing the impacts of a northern open economy on the global environment[J]. Ecological Economics, 2016, 126: 169-181.

Vandani A M K, Joda F, Boozarjomehry R B. Exergic, economic and environmental impacts of natural gas and diesel in operation of combined cycle power plants[J]. Energy Conversion and Management, 2016, 109: 103-112.

Wang Y, Chen X, Ren S. Clean energy adoption and maternal health: Evidence from China[J]. Energy Economics, 2019, 84: 104517.

Yang Z, Wei T, Moore J C, et al. A new consumption-based accounting model for greenhouse gases from 1948 to 2012[J]. Journal of Cleaner Production, 2016, 133: 368-377.

Simon J L. The economics of population growth[M]. Princeton University Press, 2019.

Springer C, Evans S, Lin J, et al. Low carbon growth in China: The role of emissions trading in a transitioning economy[J]. Applied Energy, 2019, 235: 1118-1125.

Zhong Z, Jiang L, Zhou P. Transnational transfer of carbon emissions embodied in trade: Characteristics and determinants from a spatial perspective. Energy, 2018, 147: 858-875.

Zhong Z, Zhang X, Bao Z. Spatial characteristics and driving factors of global energy-related sulfur oxides emissions transferring via international trade. Journal of Environmental Management, 2019, 249: 109370.
